# Repeated prefrontal tDCS improves cognitive emotion regulation and readiness for treatment in substance use disorder: A randomized sham-controlled study

**DOI:** 10.1016/j.abrep.2025.100614

**Published:** 2025-05-08

**Authors:** Ali Salmani, Sajjad Basharpoor, Zahra Vaziri, Mohammad Ali Salehinejad

**Affiliations:** a Department of Psychology, Faculty of Educational Sciences and Psychology, University of Mohaghegh Ardabili, Ardabil, Iran; b Department of Neuroscience and Behavior, Faculty of Medicine of Ribeirão Preto, University of São Paulo, Ribeirão Preto, Brazil; c School of Cognitive Sciences, Institute for Research in Fundamental Sciences (IPM), Tehran, Iran; d Department of Psychology and Neurosciences, Leibniz Research Centre for Working Environment and Human Factors. Dortmund, Germany; e Department of Child and Adolescent Psychiatry, Psychosomatics, and Psychotherapy, Medical Faculty, RWTH Aachen University, Aachen, Germany

**Keywords:** Dorsolateral prefrontal cortex, Emotion regulation, Substance use disorder, Transcranial direct current stimulation, Treatment motivation

## Abstract

**Background:**

Individuals with substance use disorder (iSUD) often show diminished motivation toward treatment-seeking and adherence, resulting in a higher relapse rate, especially during the abstinence phase. A key cognitive correlate of substance use and treatment-seeking behavior is the ability to regulate emotion. While transcranial direct current stimulation (tDCS) shows potential for treating SUD, most studies have focused on craving or cognitive deficits. This randomized, placebo-controlled study examines the effects of 15 daily sessions of prefrontal tDCS on treatment motivation and cognitive emotion regulation in iSUD during the abstinence phase.

**Methods:**

Thirty-two young male iSUDs (mean age= 22.80±1.92) were randomly assigned to the experimental (n=16) or placebo (n=16) groups. The experimental group underwent 15 sessions of anodal left and cathodal right dlPFC stimulation (2 mA, 20 min), while the placebo group received sham stimulation over the same period. Assessments of readiness, eagerness, motivation for treatment, and cognitive emotion regulation were conducted before and after the intervention.

**Results:**

The active repeated prefrontal tDCS significantly boosted motivation for change and treatment in iSUD in comparison to sham tDCS. This intervention specifically improved participants’ recognition of issues related to their substance use and reduced ambivalence about it. Additionally, active prefrontal tDCS enhanced adaptive cognitive emotion regulation while reducing maladaptive cognitive emotion regulation strategies in iSUD.

**Conclusions:**

Repeated prefrontal tDCS can help iSUD during the abstinence phase to improve adaptive emotion regulation strategies, thereby boosting motivation for treatment seeking. Up and downregulation of left and right dlPFCs is a promising approach for cognitively and motivationally preparing iSUD for treatment-seeking.

## Highlights

• Emotion regulation is related to craving and relapse in individuals with SUD (iSUD)
• Readiness for treatment is crucial for a successful intervention in iSUD.
• 15 sessions of prefrontal tDCS increased readiness and motivation for treatment in iSUD.
• The intervention also improved cognitive emotion regulation strategies in iSUD.
• Enhanced cognitive emotion regulation and treatment readiness were correlated.

## Graphical abstract

**Figure:**
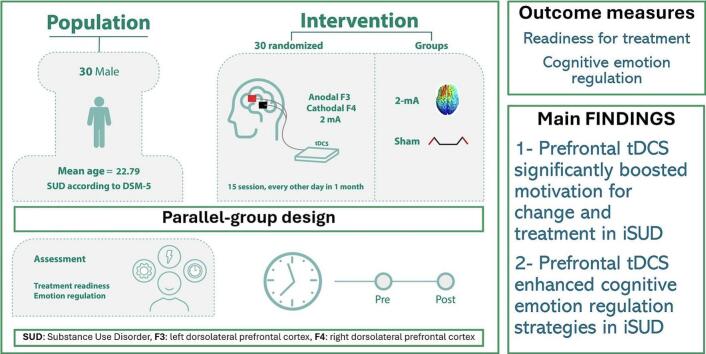

## 1 Introduction

Substance Use Disorder (SUD) is a chronically relapsing disorder with a high global burden of disease (Ferrari et al., 2024) marked by profound motivational disturbances and an inability to regulate behavior (Self & Staley, 2010). The behaviors linked to SUD stem from a complex and varied etiology, particularly with respect to the interplay between cognition, emotion, and behavior (Koob & Volkow, 2010). The DSM-5 identifies SUD as involving various cognitive, behavioral, motivational, and physiological deficits that adversely impact decision-making, treatment-seeking behavior, and treatment adherence (American Psychiatric Association, 2013).

A major challenge in treating individuals with substance use disorder (iSUD) is their limited motivation for treatment, leading to relapses and high recurrence rates, especially during the abstinence stage of addiction (Andersson et al., 2019, Kabisa et al., 2021). Previous studies highlight the importance of treatment *motivation* across all stages of substance abuse (DiClemente, 1999, Sjoerds et al., 2014), linking readiness to change behaviors with successful interventions (Miller & Rollnick, 2003). Effective treatment begins by addressing the desire for change, given that iSUD are often treatment-seeking resistant and show lower motivation for treatment (Gressler et al., 2019). The stages of treatment motivation in SUD include *recognition*, *ambivalence*, and *action* phases, and the complex interplay between cognition-emotion is involved in all phases (Miller & Tonigan, 1996).

One important factor of treatment motivation is emotion regulation, which is often impaired in iSUD (Contreras-Rodríguez et al., 2020, Stellern et al., 2023). In SUD, effective emotional management can mitigate drug cravings, whereas poor regulation may predict treatment failure (Sayette, 2016, Wills et al., 2017). Emotion regulation has cognitive components that are critical for managing emotional processing to support adaptive coping behaviors in high-risk situations (Gili et al., 2017, Gressler et al., 2019). On the other hand, poor emotion regulation impairs cognitive functions (Stellern et al., 2023), creating a cycle of dysregulation that drives craving and relapse. The bidirectional interaction between cognition and emotion in SUD is tightly linked through prefrontal-limbic circuits (Koob & Volkow, 2010).

The prefrontal cortex is a key cortical region for cognitive control (Salehinejad et al., 2021, Salehinejad et al., 2021) and contributes to cravings for cigarettes (McBride et al., 2006), alcohol (Tapert et al., 2003), and SUD including opioids (Rosen et al., 2015), cocaine (Chen et al., 2013) and methamphetamine (Alizadehgoradel et al., 2020). It also plays a role in cognitive emotion regulation (Ochsner et al., 2012, Salehinejad et al., 2021, Salehinejad et al., 2021) and its activation can influence limbic subcortical regions associated with reward and punishment processing (Alizadehgoradel et al., 2020, Molavi et al., 2020, Vicario et al., 2019). Transcranial direct current stimulation (tDCS) is a safe and easy-to-use non-invasive brain stimulation (NIBS) intervention for studying and modifying human brain functions (Polanía et al., 2018, Salehinejad and Siniatchkin, 2024) with therapeutic promise for brain disorders with altered activation in the prefrontal cortex (Begemann et al., 2020, Li et al., 2022, Salehinejad et al., 2022).

**Fig. 1:**
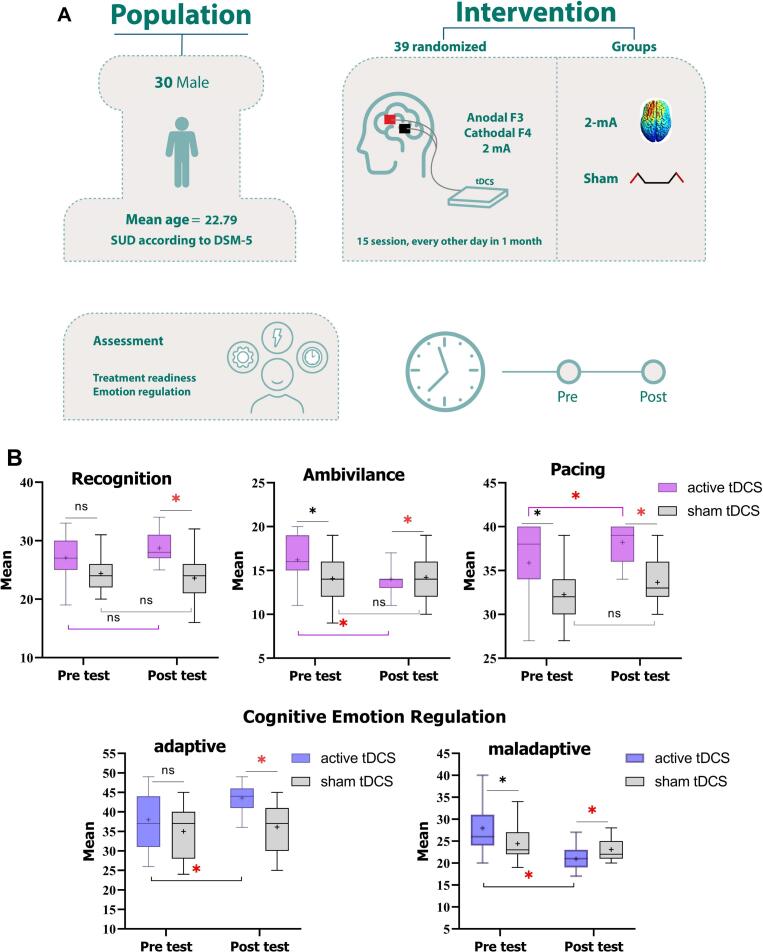
**A**. Experimental procedure. This was a randomized, sham-controlled study on 30 male individuals with substance use disorder. *Note*: the electrode positions are schematic and do not represent actual placements. The electrodes were applied to the left dorsolateral prefrontal cortex (F3) for the anode and the right dorsolateral prefrontal cortex for the cathode. **B**. The impact of bilateral dlPFC tDCS on treatment motivation and cognitive emotion regulation. Red asterisks indicate that the active tDCS group showed significant improvements in recognition, pacing, and adaptive emotion regulation strategies, along with a reduction in treatment ambivalence and maladaptive emotional regulation. Black asterisks represent baseline value differences. *Note*: ns: nonsignificant, tDCS: transcranial direct current stimulation.

There is a growing interest in tDCS interventions for treating drug-related disorders (Coles et al., 2018, Mehta et al., 2024). While NIBS studies have shown promise in addressing cognitive impairments and cravings in iSUD (Nakamura-Palacios et al., 2021, Stein et al., 2019, Zhang et al., 2024), the impact of tDCS on motivation for change, treatment readiness, and cognitive-emotional control remains largely unknown, with few studies having examined emotion regulation in iSUD using tDCS (da Silva et al., 2013, Zoghipaydar et al., 2022). Therefore, this study investigates the effects of up- and downregulation of the dorsolateral prefrontal cortex (dlPFC) via tDCS on (1) treatment motivation and (2) cognitive emotion regulation in abstinent iSUD individuals. This phase is particularly critical due to heightened vulnerability to relapse, which is also associated with altered dlPFC activation Goldstein & Volkow, 2011. We also explored (3) the relationships between treatment-seeking motivation, cognitive emotion regulation, and craving. It is noteworthy that this study is among the very few tDCS studies that applied 15 sessions of tDCS in SUD (Zhang et al., 2024) and has thus important implications for the efficacy of longer periods of tDCS sessions for addiction medicine (see Fig. 1).

## 2 Methods

### 2.1 Participants

Participants were selected from the Nejat Abstinence Center in Ardabil, Iran. The sample size was calculated using power analysis based on a medium to large effect size and also previous studies (Mehta et al., 2024, Zhang et al., 2024), resulting in 32 individuals (f = 0.30 equal to η_p_^2^ 0.085, α = 0.05, power = 0.90, mixed-model ANOVA). Sixty-two iSUD, during the abstinence phase (post-detoxification), were initially screened for inclusion, with thirty individuals excluded for not meeting the inclusion criteria (see supplementary information for CONSORT diagram). This was to prevent from potential confounding effect of treatment seeking due to the withdrawal stage. Thirty-two participants who met the inclusion criteria were randomly assigned [1:1 ratio] to either the experimental group (n = 16) or the control group (n = 16). Two individuals did not complete the study—one due to leaving the center and another due to discomfort during tDCS, resulting in a final analysis of 30 participants (see the CONSORT flowchart in the supplementary material). Inclusion criteria were: (1) age 18–25, WHO-identified high-risk group for SUD (Drugs & Crime, 2018); (2) DSM-5 SUD diagnosis; (3) absence of intracranial implants/nearby metal; (4) no history of epilepsy, brain injury, or neurological disorders. All participants were recruited from abstinence centers and rigorously monitored for substance use throughout the experiment. Aside from tDCS, no other treatment was administered. The study was approved by the Ethics Committee of the University of Mohaghegh Ardabili (Ethics code: IR.UMA.REC.1400.003). All participants provided written informed consent and were free to withdraw at any time (Demographics are in Table 1).

**Table 1:** Demographic information.

**Variable**		**active tDCS**	**sham tDCS**	**sig**
Sample size		15	15	n/a
Age – Mean (SD)		22.33 (2.58)	23.26 (1.09)	0.133
Sex – Male (female)		15 (0)	15 (0)	n/a
Marital status	Single	10	6	0.028
	Married	4	7	
	Divorced	1	2	
Employment	Unemployed	5	2	0.189
	Self-employed	10	11	
	Employee	0	2	
Education	Under diploma	7	7	0.319
	Diploma or higher	8	8	
SUD history-Mean (SD)	Years	4.46 (2.64)	4.60 (2.32)	0.884
Substance type	Cannabis	2	2	0.852
	Methamphetamine	4	3	
	Opioid	5	3	
	Cocaine	1	3	
	Heroin	1	3	
	Marijuana	2	2	
Quitting attempt	Once	6	6	0.999
	Twice	5	5	
	Triple and more	4	4	
Substance use method	Inhalation	8	4	0.388
	Injection	0	1	
	Ingestion	2	2	
	Snorting	5	8	

*Note*: SUD = Substance Use Disorder; M = Mean; SD = Standard Deviation; n/a = not applicable. Between-group differences in demographic variables were explored by Chi-square tests or Fisher's exact test for categorical variables and *F*-tests for continuous variables.

### 2.2 Measures: treatment eagerness and cognitive emotion regulation

The Stages of Change Readiness and Treatment Eagerness Scale (SOCRATES) (Miller & Tonigan, 1996) was used to measure readiness to change and eagerness for treatment, particularly in the context of substance abuse and addiction. It consists of 19 questions across three subscales: recognition, ambivalence, and pacing, with Cronbach's alpha coefficients ranging from 0.60 to 0.96 across the subscales (Miller & Tonigan, 1996). Additionally, the Cognitive Emotion Regulation Questionnaire (CERQ) (Garnefski & Kraaij, 2007) was used to measure cognitive emotion regulation in response to stressful or challenging situations. It categorizes cognitive regulation into adaptive and maladaptive strategies, each with various subscales. Adaptive strategies include acceptance, putting into perspective, positive reappraisal, and planning, while maladaptive strategies include self-blame, other-blame, rumination, and catastrophizing. A higher score indicates greater use of cognitive strategies for emotion regulation. Participants' craving ratings were also assessed and reported in a previous work (Salmani et al., 2024). A detailed description of measures is in the supplementary information.

### 2.3 tDCS

We used a two-channel Neurostim stimulator (MadinaTeb, Tehran, Iran) powered by a 9-volt alkaline battery. In active tDCS, electrical current was applied through rubber electrodes positioned between saline-soaked sponges (25 cm^2^) for 20 min, including a 30-second ramp-up and ramp-down, at 2 mA intensity. The stimulation protocol was adopted from our previous work (Alizadehgoradel et al., 2020) although stimulation protocols used in SUD vary in parameters (Zhang et al., 2024). Participants in the experimental group underwent real tDCS for 15 daily sessions with anodal and cathodal electrodes placed over the left (F3) and right dlPFC (F4) respectively, according to the 10–20 international EEG system. The daily tDCS session is based on the guidelines for addiction medicine (Ekhtiari et al., 2019) and previous clinical tDCS studies (Fregni et al., 2020). The 15-session regimen was chosen to evaluate the effectiveness of a longer tDCS protocol compared to the typical 5 or 10 sessions (Gaudreault et al., 2021). In the sham condition, the electrical current was ramped up for 30 s, followed by 30 s of stimulation and ramped down for 30 s to mimic the sensation of the active condition. Participants were unaware of the type of stimulation they received. A side-effect survey was carried out after each session to record any reported side effects (Brunoni et al., 2011), but blinding efficacy was not investigated due to the multi-session design of the study. The experimenter was not blind to the stimulation conditions.

### 2.4 Procedure

First, participants provided informed consent and completed a brief questionnaire to assess their suitability for brain stimulation. Both the active and sham tDCS groups underwent 15 stimulation sessions with 24-hour intervals between sessions. The SOCRATES and CERQ measures were assessed before the first and after the final tDCS session. tDCS sessions were scheduled at fixed times of the day for each subject, ensuring no sleep pressure to minimize the potential impact of circadian variations on cortical excitability and neuroplasticity induction throughout all sessions (Salehinejad et al., 2022; Salehinejad et al., 2021). Patients in the sham group received active tDCS intervention at the end of the study.

### 2.5 Statistical analysis

Data was analyzed with the statistical package SPSS (V 26, IBM, SPSS, Inc., Chicago, IL) and the GraphPad Prism (GraphPad Software, San Diego, California). The normality and homogeneity of data distribution were confirmed by Shapiro-Wilk and Levin tests. We observed differences in pre-test scores of some variables and accordingly, a multivariate analysis of covariance (MANCOVA) was conducted on post-intervention outcome measures with group (active vs sham) as the fixed factor and pre-intervention measures as covariates. This is the suggested method when there is a pre-test difference (Van Breukelen, 2006) to control for baseline differences in outcome measures. The M-box test was used to assess the equality of covariance matrices across groups before conducting the MANCOVA. In case of significant results, post hoc ANCOVAs were conducted to determine which dependent variables were significantly different between the two groups. Effect sizes are reported in partial et squared (η^2^) with values of 0.01, 0,06, and 0.14 corresponding to small, medium and large effects, respectively. Pairwise comparisons were then conducted using Bonferroni-corrected t-tests. Correlational analyses were conducted to determine whether changes in outcome variables are related to each other and participants' craving scores. The critical level of significance was 0.05 for all statistical analyses.

## 3 Results

### 3.1 Data overview

Participants well-tolerated the stimulation, and no adverse effects were reported during and after stimulation. One participant withdrew from the study due to uncomfortable sensations during the stimulation. Between-group ratings of tDCS side effects were not significantly different. No significant differences were observed between-group demographics, including the types of SUD, except for marital status. Baseline scores of the outcome variables are shown in Table S3.

### 3.2 Efficacy of bilateral dlPFC tDCS on readiness for treatment and cognitive emotion regulation

The results of the MANCOVA conducted on the post-intervention scores of outcome measures (SOCRATES, CERQ) showed a significant main effect of group on at least one outcome measure after controlling for baseline differences (*F_19_* = 15.60, *p* < 0.001, *η_p_^2^* = 0.99, Wilk's Lambda = 0.005). The follow-up ANCOVAs showed a significant main effect of the group on subscales of SOCRATES including recognition (*F*_1_ = 5.12, *p* = 0.033, *η_p_^2^* = 0.18), ambivalence (*F*_1_ = 12.73, *p* = 0.002, *η_p_^2^* = 0.356), and pacing (*F_1_* = 13.82, *p* < 0.001, *η_p_^2^* = 0.375). Bonferroni-corrected post hoc t-tests revealed that the active tDCS significantly increased recognition (*p* = 0.013) and pacing (*p* < 0.001) in the group that received active tDCS while reducing their ambivalence (*p* = 0.008) toward treatment. Similarly, a significant main effect of group was observed on adaptive (*F_1_* = 24.58, *p* < 0.001, *η_p_^2^* = 0.517) and maladaptive (*F_1_* = 4.31, *p* = 0.049, *η_p_^2^* = 0.158) emotion regulation. Bonferroni-corrected post hoc t-tests revealed that the active tDCS enhanced adaptive (*p* < 0.001) and reduced maladaptive (*p* = 0.019) emotion regulation strategies in iSUD. Furthermore, Pearson’s correlational analysis indicated that improved adaptive cognitive-emotional regulation was significantly correlated with increased treatment motivation, specifically in recognition (*r* = 0.559, *p* < 0.001) and pacing (*r* = 0.681, *p* < 0.001). In a separate publication, we reported that craving scores were significantly lower in the active tDCS group (mean = 1.46 ± 1.95) but not in the sham (mean = 3.40 ± 3.39) (Salmani et al., 2024). We did a similar correlation analysis between treatment motivation and craving scores of participants and found that treatment motivation was significantly associated with reduced craving (*r_adaptive_* = −0.568, *p* < 0.001; *r_recognition_* = −80.323, *p* = 0.042; *r_stepping_* = −0.413, *p* = 0.023).

## 4 Discussion

Readiness for treatment is the primary step in addressing substance-related addictive disorders. In this randomized sham-controlled study, 15 sessions of anodal left-cathodal right dlPFC tDCS, improved cognitive emotion regulation strategies and at the same time increased readiness for treatment in young iSUD. Importantly, improvement in both measures was significantly associated. To the best of our knowledge, this has not been studied in previous NIBS studies of SUD. We briefly discuss the neurophysiological mechanism behind the observed effect and its association with outcome measures.

One potential mechanism for the observed effects of our prefrontal tDCS intervention on iSUDs involves the dlPFC's role in reducing negative emotions and enhancing positive ones, which can encourage treatment-seeking. The dlPFC is crucial for the cognitive control of emotion processing (Nejati et al., 2021, Ochsner et al., 2012, Salehinejad et al., 2021, Salehinejad et al., 2021), with the left one associated with positive emotions and the right with negative ones (Alves et al., 2008, Canli et al., 1998). The left dlPFC uses top-down processes to modulate emotional processing regions, allowing individuals to manage their emotional responses and maintain balance during emotional challenges (Ochsner et al., 2012). Conversely, inhibitory stimulation of the right dlPFC amplifies attention to negative stimuli and reduces cognitive control (De Raedt et al., 2010). The altered activation of prefrontal dopamine, linked to impaired decision-making and reward valuation in SUD (Goldstein & Volkow, 2011) and association of tDCS neuroplasiticty-induced effects with dopamine modulation (Ghanavati et al., 2025, Ghanavati, Salehinejad, De Melo, Nitsche, & Kuo, 2022) is likely another explanation. Furthermore, the role of dlPFC anodal stimulation in regulating positive and negative emotions is seen in SUD and other emotional disorders too (Jafari et al., 2021, Nikolin et al., 2023, Vicario et al., 2019, da Silva et al., 2013). This, together with the contribution of dlPFC to improved executive control in healthy and clinical populations (Ghanavati et al., 2019, Narmashiri and Akbari, 2023, Salehinejad et al., 2017, Zakibakhsh et al., 2024), including SUD (Alizadehgoradel et al., 2020, Zhang et al., 2024), might explain increased treatment motivation and enhanced adaptive cognitive emotion regulation.

In addition to the hypothesized mechanism of effect, it's essential to evaluate how increased treatment motivation and improved emotion regulation impact treatment-related outcomes (e.g., craving, relapse). This intervention also reduced the cravings of participants (Salmani et al., 2024), in line with our earlier work on methamphetamine use disorder (Alizadehgoradel et al., 2020). However, examining whether enhanced treatment motivation prevented relapse was not feasible since participants were in an abstinence center. Nonetheless, increased motivation for change is a major predictor of recovery from substance use, as shown in a longitudinal study (DiClemente, 1999), and is expected to correlate with meaningful changes in substance use behavior. Future studies are needed to objectively assess how the change in treatment motivation affects SUD.

In conclusion, up- and downregulation of left and right dlPFC, respectively, could be a promising intervention to encourage treatment seeking and treatment adherence in young iSUD by enhancing emotion regulation and reducing craving. Limitations of this study were the inclusion of only male participants, lack of control over the type of substance used, absence of behavioral and physiological outcome measures, no investigation of blinding efficacy in participants, and constraints imposed by the COVID-19 pandemic, including lack of follow-up assessment. Lastly, this study targeted individuals with iSUD in abstinence centers, either seeking treatment or needing to abstain from SUD. To effectively evaluate changes in motivation for seeking and adhering to treatment after tDCS, a group of non-treatment-seeking individuals with iSUD is necessary.

## CRediT authorship contribution statement

**Ali Salmani:** Validation, Investigation, Formal analysis, Data curation. **Sajjad Basharpoor:** Supervision, Resources, Project administration, Methodology, Conceptualization. **Zahra Vaziri:** Writing – review & editing, Visualization. **Mohammad Ali Salehinejad:** Writing – review & editing, Writing – original draft, Visualization, Supervision, Methodology.

## Funding

None.

## Declaration of competing interest

The authors declare that they have no known competing financial interests or personal relationships that could have appeared to influence the work reported in this paper.

## Data Availability

Data will be made available on request.
