# Supplementary material for: Repeated prefrontal tDCS improves cognitive emotion regulation and readiness for treatment in substance use disorder: A randomized sham-controlled study
Source: Addict Behav Rep. 2025 May 8;21:100614. doi: 10.1016/j.abrep.2025.100614 (PMC12136907; doi:10.1016/j.abrep.2025.100614)
Supplement: Supplementary Data 1 [file mmc1.docx]

**Methods**

***Consort diagram of study inclusion***

|  |
| --- |
| Figure S1: CONSORT flowchart of study inclusion |

***Measures***

*2.2.1. The Stages of Change Readiness and Treatment Eagerness Scale (**SOCRATES)*

This scale was made by (Miller and Tonigan, 1996) to measure the readiness and acceptance for change and treatment of people with substance-related addiction disorders. It comprises 19 questions across three subscales: recognition, ambivalence, and pacing (or taking steps). The recognition subscale measures the individual's acknowledgment of problems associated with their substance use. Higher scores indicate greater awareness of the negative consequences of their behavior and a willingness to consider the need for change. The ambivalence subscale reflects the individual's mixed feelings about their substance use. It captures the conflict between wanting to change and wanting to continue the behavior, indicating a struggle with motivation. The pacing subscale assesses the extent to which the individual has initiated steps toward change. It focuses on the actions taken to modify behavior, such as seeking help or modifying substance use habits, showing proactivity in the change process. Strong correlations were observed with a 39-question variant. Cronbach's alpha coefficients ranged from 0.70 to 0.90 across the subscales (Miller and Tonigan, 1996; Tonigan et al., 1997). The Chronbach’s alphas of 0.76, 0.71, and 0.85 for the recognition, ambivalence, and pacing subscales are reported in the native-language version of the scale (Basharpoor, 2016).

*2.2.2. Cognitive Emotion Regulation Questionnaire (CERQ)*

This questionnaire was developed by (Garnefski and Kraaij, 2007) and is a self-reported instrument. It is a psychological assessment tool used to measure individuals' cognitive strategies for regulating emotions in response to stressful or challenging situations. It measures individuals' cognitive strategies for managing emotions during stressful situations. The short form comprises 18 items assessing cognitive strategies in response to risky and stressful events on a five-point scale across 9 subscales. Scores range from 1 ("never") to 5 ("always"). The test categorizes cognitive regulation into adaptive and maladaptive types, each with various subscales. Adaptive strategies include acceptance, putting into perspective, positive reappraisal, and planning, while maladaptive strategies include self-blame, other-blame, rumination, and catastrophizing. A higher score indicates greater use of cognitive strategies for emotion regulation. Internal consistency is generally high, with Cronbach's alpha values typically above 0.70 for its various subscales. Validation of the questionnaire in the native language shows a Cronbach's alpha coefficient of 0.95 and 0.88 for adaptive and maladaptive subscales (Mohsenabadi and Fathi-Ashtiani, 2021).

**Supplementary Tables**

Table S2. Mean and standard deviation of tDCS side effects

|  | |  | **active tDCS** | **sham tDCS** |  |  |
| --- | --- | --- | --- | --- | --- | --- |
|  | | **sensation** | **M (SD)** | **M (SD)** | ***F*** | ***p*-value** |
| **Reported side effect** | Itching | |  |  |  |  |
|  |  |  | 0.284 (0.406) | 0.245 (0.398) | 0.31 | 0.695 |
|  |  | |  |  |  |  |
|  | Burning | | 0.895 (0.580) | 0.780 (0.532) | 0.42 | 0.624 |
|  |  | |  |  |  |  |
|  | Pain | | 0.210 (0.426) | 0.201 (0.412) | 0.16 | 0.823 |
|  |  | |  |  |  |  |
|  | Skin redness | | 0.361 (0.428) | 0.126 (0.324) | 0.98 | 0.395 |
|  |  | |  |  |  |  |
|  | Fatigue | | 0.426 (0.640) | 0.305 (0.586) | 0.21 | 0.768 |
|  |  | |  |  |  |  |
|  | Trouble concentrating | | 0.287 (0.458) | 0.065 (0.345) | 1.25 | 0.332 |

Values are presented as means ± standard deviation (SD). *Note:* each value represents the average of side effects reported during all 15 tDCS sessions. tDCS = transcranial Direct Current Stimulation; M = Mean; SD = Standard Deviation; (*p* ≤ 0.05)

Table S3. Mean and standard deviation of outcome measures

| **Measure** | **Outcome**  **variable** | **Time** | **active tDCS** | **sham tDCS** | ***p*-value** |
| --- | --- | --- | --- | --- | --- |
|  |  |  | **M (SD)** | **M (SD)** |  |
| **SOCRATES** | recognition | Pre-intervention | 27.06 (3.73) | 24.40 (2.99) | 0.022 |
|  |  | Post-intervention | 28.73 (2.54) | 23.60 (3.96) |  |
|  |  |  |  |  |  |
|  | ambivalence | Pre-intervention | 16.00 (2.70) | 14.06 (3.01) | 0.042 |
|  |  | Post-intervention | 14.00 (1.51) | 14.20 (2.67) |  |
|  |  |  |  |  |  |
|  | pacing | Pre-intervention | 35.86 (4.20) | 32.26 (3.28) | 0.247 |
|  |  | Post-intervention | 38.20 (1.89) | 33.66 (2.58) |  |
|  |  |  |  |  |  |
| **Cognitive emotion regulation** | adaptive | Pre-intervention | 38.00 (7.30) | 35.00 (7.04) | 0.262 |
|  |  | Post-intervention | 43.53 (3.48) | 36.13 (6.08) |  |
|  |  |  |  |  |  |
|  | maladaptive | Pre-intervention | 27.93 (5.32) | 24.40 3.97) | 0.065 |
|  |  | Post-intervention | 20.93 (2.68) | 23.06 (2.46) |  |

*Note*: tDCS = transcranial Direct Current Stimulation; M = Mean; SD = Standard Deviation; SOCRATES = The Stages of Change Readiness and Treatment Eagerness Scale. *p* values refer to between-group baseline (i.e., pre-intervention) comparisons by ANOVA tests.

**Table 4**: Multivariate analysis of covariance test

| **Effect** | **Value** | **F** | **Hypothesis df** | **Error df** | **Sig.** | **Observed Power** |
| --- | --- | --- | --- | --- | --- | --- |
| Pillai's Trace | 0.80 | 15.60 | 5.00 | 19.00 | 0.001 | 0.995 |
| Wilks' Lambda | 0.19 | 15.60 | 5.00 | 19.00 | 0.001 | 0. 995 |
| Hotelling's Trace | 4.10 | 15.60 | 5.00 | 19.00 | 0.001 | 0. 995 |
| Roy's Largest Root | 4.10 | 15.60 | 5.00 | 19.00 | 0.001 | 0. 995 |

**Table 5**: Tests of Between-Subjects Effects

| **Source** | **Dependent Variable** | **Sum of Squares** | **df** | **Mean Square** | **F** | **Sig.** | **Observed Power^f^** |
| --- | --- | --- | --- | --- | --- | --- | --- |
| **Pretest effect** | recognition | 56.06 | 1 | 56.06 | 6.47 | 0.018 | 0.683 |
|  | ambivalence | 64.94 | 1 | 64.94 | 42.14 | 0.001 | 1.000 |
|  | pacing | 38.87 | 1 | 38.67 | 14.43 | 0.001 | 0.953 |
|  | adaptive emotion regulation | 227.67 | 1 | 227.67 | 34.98 | 0.001 | 1.000 |
|  | maladaptive emotion regulation | 10.26 | 1 | 10.26 | 1.40 | 0.248 | 0.206 |
| **Group effect** | recognition | 44.40 | 1 | 44.40 | 5.12 | 0.033 | 0.583 |
|  | ambivalence | 19.63 | 1 | 19.63 | 12.73 | 0.002 | 0.927 |
|  | pacing | 37.05 | 1 | 37.05 | 13.82 | 0.001 | 0.945 |
|  | adaptive emotion regulation | 160.00 | 1 | 160.00 | 24.58 | 0.000 | 0.997 |
|  | maladaptive emotion regulation | 20.02 | 1 | 31.43 | 4.31 | 0.049 | 0.512 |

**References**

Basharpoor S (2016) The Effectiveness of Cognitive-Behavioral Coping Skills Training and Mindfulness-Based Relapse Prevention Program in the Improvement of Treatment Motivation in Drug Dependent Individuals. etiadpajohi 10:55-70.

Garnefski N, Kraaij V (2007) The cognitive emotion regulation questionnaire. European journal of psychological assessment 23:141-149.

Miller WR, Tonigan JS (1996) Assessing drinkers' motivation for change: The Stages of Change Readiness and Treatment Eagerness Scale (SOCRATES). Psychol Addict Behav 10:81-89.

Mohsenabadi H, Fathi-Ashtiani A (2021) Evaluation of psychometric properties of the Persian version of the short form of Cognitive Emotion Regulation Questionnaire (CERQ-18). Payesh (Health Monitor) Journal 20:167-178.

Tonigan JS, Miller WR, Brown JM (1997) The reliability of Form 90: an instrument for assessing alcohol treatment outcome. J Stud Alcohol 58:358-364.
